# Supplementary material for: Novel Machine Learning Approach to Predict and Personalize Length of Stay for Patients Admitted with Syncope from the Emergency Department
Source: J Pers Med. 2022 Dec 20;13(1):7. doi: 10.3390/jpm13010007 (PMC9864075; doi:10.3390/jpm13010007)
Supplement: Supplementary file 1 [file jpm-13-00007-s001.zip › jpm-1971927-supplementary.pdf]

## Table S1: Disposition of the Patient Population.

| | Total (n = 4,645,483) | | 2016 (n = 1,145,359) | | 2017 (n = 1,174,452) | | 2018 (n = 1,137,276) | | 2019 (n = 1,188,396) | | *p*-Value |
|---|---|---|---|---|---|---|---|---|---|---|---|
| Disposition of patient (uniform) from ED | | | | | | | | | | | |
| Routine | 3,881,001 | (83.5%) | 952,763 | (83.2%) | 973,924 | (82.9%) | 951,524 | (83.7%) | 1,002,790 | (84.4%) | 0.0246 |
| Transfer to short-term hospital | 95,784 | (2.1%) | 25,467 | (2.2%) | 23,552 | (2.0%) | 22,297 | (2.0%) | 24,468 | (2.1%) | |
| Skilled Nursing Facility (SNF) | 81,749 | (1.8%) | 19,368 | (1.7%) | 20,959 | (1.8%) | 20,017 | (1.8%) | 21,406 | (1.8%) | |
| Home Health Care (HHC) | 54,042 | (1.2%) | 11,299 | (1.0%) | 14,061 | (1.2%) | 13,768 | (1.2%) | 14,914 | (1.3%) | |
| Against medical advice (AMA) | 156,651 | (3.4%) | 37,105 | (3.2%) | 41,714 | (3.6%) | 37,099 | (3.3%) | 40,733 | (3.4%) | |
| Admitted inpatient to this hospital | 374,947 | (8.1%) | 98,894 | (8.6%) | 99,912 | (8.5%) | 92,344 | (8.1%) | 83,798 | (7.1%) | |
| Died in ED | 1,309 | (0.0%) | 464 | (0.0%) | 330 | (0.0%) | 227 | (0.0%) | 288 | (0.0%) | |
| Disposition of patient (uniform) from IP | | | | | | | | | | | |
| Routine | 245,120 | (65.4%) | 65,759 | (66.5%) | 65,099 | (65.2%) | 60,044 | (65.0%) | 54,219 | (64.7%) | 0.0253 |
| Transfer to short-term hospital | 5,560 | (1.5%) | 1,461 | (1.5%) | 1,458 | (1.5%) | 1,423 | (1.5%) | 1,218 | (1.5%) | |
| Skilled Nursing Facility (SNF) | 56,198 | (15.0%) | 15,302 | (15.5%) | 14,941 | (15.0%) | 13,488 | (14.6%) | 12,467 | (14.9%) | |
| Home Health Care (HHC) | 54,554 | (14.5%) | 12,857 | (13.0%) | 14,968 | (15.0%) | 13,883 | (15.0%) | 12,846 | (15.3%) | |
| Against medical advice (AMA) | 12,586 | (3.4%) | 3,227 | (3.3%) | 3,211 | (3.2%) | 3,304 | (3.6%) | 2,844 | (3.4%) | |
| Died in ED | 929 | (0.2%) | 287 | (0.3%) | 235 | (0.2%) | 203 | (0.2%) | 204 | (0.2%) | |

## Table S2: Hospital Demographics.

| | Total (n = 4,645,483) | | 2016 (n = 1,145,359) | | 2017 (n = 1,174,452) | | 2018 (n = 1,137,276) | | 2019 (n = 1,188,396) | | *p*-Value |
|---|---|---|---|---|---|---|---|---|---|---|---|
| Control/ownership of the hospital | | | | | | | | | | | |
| Government or private | 2,017,771 | (43.4%) | 819,639 | (71.6%) | 862,422 | (73.4%) | 159,803 | (14.1%) | 175,906 | (14.8%) | |
| Government, nonfederal | 377,271 | (8.1%) | 52,146 | (4.6%) | 53,784 | (4.6%) | 135,361 | (11.9%) | 135,979 | (11.4%) | |
| Private, not-for-profit | 1,430,591 | (30.8%) | 149,819 | (13.1%) | 140,108 | (11.9%) | 503,342 | (44.3%) | 637,322 | (53.6%) | < 0.001 |
| Private, investor-owned | 360,767 | (7.8%) | 64,161 | (5.6%) | 63,181 | (5.4%) | 118,161 | (10.4%) | 115,263 | (9.7%) | |
| Private | 459,084 | (9.9%) | 59,593 | (5.2%) | 54,957 | (4.7%) | 220,609 | (19.4%) | 123,925 | (10.4%) | |
| Region of hospital | | | | | | | | | | | |
| Northeast | 935,245 | (20.1%) | 225,265 | (19.7%) | 237,677 | (20.2%) | 233,475 | (20.5%) | 238,827 | (20.1%) | |
| Midwest | 1,034,363 | (22.3%) | 259,122 | (22.6%) | 258,084 | (22.0%) | 249,113 | (21.9%) | 268,044 | (22.6%) | 1 |
| South | 1,752,100 | (37.7%) | 429,525 | (37.5%) | 450,707 | (38.4%) | 425,176 | (37.4%) | 446,693 | (37.6%) | |
| West | 923,775 | (19.9%) | 231,447 | (20.2%) | 227,985 | (19.4%) | 229,512 | (20.2%) | 234,832 | (19.8%) | |
| Teaching status of the hospital | | | | | | | | | | | |
| Metropolitan non-teaching | 1,140,700 | (24.6%) | 335,392 | (29.3%) | 302,280 | (25.7%) | 260,522 | (22.9%) | 242,505 | (20.4%) | |
| Metropolitan teaching | 2,835,167 | (61.0%) | 642,257 | (56.1%) | 707,586 | (60.2%) | 708,181 | (62.3%) | 777,143 | (65.4%) | < 0.001 |
| Non-metropolitan hospital | 669,616 | (14.4%) | 167,710 | (14.6%) | 164,587 | (14.0%) | 168,572 | (14.8%) | 168,747 | (14.2%) | |
| Hospital urban-rural designation | | | | | | | | | | | |
| Large metropolitan | 2,434,656 | (52.4%) | 594,776 | (51.9%) | 614,437 | (52.3%) | 599,271 | (52.7%) | 626,171 | (52.7%) | |
| Small metropolitan | 1,468,398 | (31.6%) | 364,988 | (31.9%) | 378,345 | (32.2%) | 353,976 | (31.1%) | 371,089 | (31.2%) | |
| Micropolitan areas | 379,372 | (8.2%) | 91,020 | (7.9%) | 88,808 | (7.6%) | 94,662 | (8.3%) | 104,881 | (8.8%) | < 0.001 |
| Not metropolitan or micropolitan | 207,053 | (4.5%) | 52,333 | (4.6%) | 51,996 | (4.4%) | 51,852 | (4.6%) | 50,872 | (4.3%) | |

## Table S3: Elixhauser Comorbidity Indices of the Patient Population.

| | Total (n = 4,645,483) | | 2016 (n = 1,145,359) | | 2017 (n = 1,174,452) | | 2018 (n = 1,137,276) | | 2019 (n = 1,188,396) | | *p*-Value |
|---|---|---|---|---|---|---|---|---|---|---|---|
| Hypertension, Uncomplicated | 1,522,125 | (32.8%) | 396,318 | (34.6%) | 388,954 | (33.1%) | 362,781 | (31.9%) | 374,071 | (31.5%) | < 0.001 |
| Cardiac Arrhythmias | 720,176 | (15.5%) | 166,000 | (14.5%) | 180,669 | (15.4%) | 179,898 | (15.8%) | 193,608 | (16.3%) | < 0.001 |
| Fluid and Electrolyte Disorders | 672,863 | (14.5%) | 160,183 | (14.0%) | 167,846 | (14.3%) | 171,049 | (15.0%) | 173,785 | (14.6%) | 0.0228 |
| Diabetes, Uncomplicated | 484,394 | (10.4%) | 130,952 | (11.4%) | 123,531 | (10.5%) | 112,420 | (9.9%) | 117,489 | (9.9%) | < 0.001 |
| Chronic Pulmonary Disease | 444,555 | (9.6%) | 105,206 | (9.2%) | 112,837 | (9.6%) | 112,663 | (9.9%) | 113,849 | (9.6%) | 0.0801 |
| Hypertension, Complicated | 373,160 | (8.0%) | 63,601 | (5.6%) | 94,545 | (8.1%) | 101,050 | (8.9%) | 113,964 | (9.6%) | < 0.001 |
| Hypothyroidism | 320,596 | (6.9%) | 76,431 | (6.7%) | 82,281 | (7.0%) | 80,053 | (7.0%) | 81,831 | (6.9%) | 0.2352 |
| Renal Failure | 299,530 | (6.4%) | 66,991 | (5.8%) | 74,820 | (6.4%) | 74,973 | (6.6%) | 82,746 | (7.0%) | < 0.001 |
| Depression | 268,992 | (5.8%) | 60,975 | (5.3%) | 69,875 | (5.9%) | 68,603 | (6.0%) | 69,539 | (5.9%) | 0.0106 |
| Congestive Heart Failure | 264,362 | (5.7%) | 58,661 | (5.1%) | 66,339 | (5.6%) | 67,155 | (5.9%) | 72,207 | (6.1%) | < 0.001 |
| Diabetes, Complicated | 248,525 | (5.3%) | 44,187 | (3.9%) | 63,610 | (5.4%) | 66,211 | (5.8%) | 74,518 | (6.3%) | < 0.001 |
| Other Neurological Disorders | 231,279 | (5.0%) | 55,258 | (4.8%) | 58,384 | (5.0%) | 56,663 | (5.0%) | 60,974 | (5.1%) | 0.1529 |
| Obesity | 184,258 | (4.0%) | 37,956 | (3.3%) | 44,553 | (3.8%) | 49,279 | (4.3%) | 52,471 | (4.4%) | < 0.001 |
| Valvular Disease | 165,882 | (3.6%) | 38,815 | (3.4%) | 41,473 | (3.5%) | 42,332 | (3.7%) | 43,263 | (3.6%) | 0.3366 |
| Peripheral Vascular Disorders | 146,736 | (3.2%) | 36,553 | (3.2%) | 36,316 | (3.1%) | 36,236 | (3.2%) | 37,630 | (3.2%) | 0.9179 |
| Drug Abuse | 131,533 | (2.8%) | 29,553 | (2.6%) | 32,686 | (2.8%) | 34,756 | (3.1%) | 34,538 | (2.9%) | 0.0006 |
| Alcohol Abuse | 113,412 | (2.4%) | 29,852 | (2.6%) | 27,780 | (2.4%) | 28,033 | (2.5%) | 27,748 | (2.3%) | 0.003 |
| Rheumatoid Arthritis/Collagen Vascular | 60,798 | (1.3%) | 13,813 | (1.2%) | 15,762 | (1.3%) | 15,242 | (1.3%) | 15,981 | (1.3%) | 0.0162 |
| Solid Tumor Without Metastasis | 57,704 | (1.2%) | 13,869 | (1.2%) | 14,246 | (1.2%) | 14,016 | (1.2%) | 15,573 | (1.3%) | 0.1828 |
| Deficiency Anemia | 49,834 | (1.1%) | 11,295 | (1.0%) | 12,392 | (1.1%) | 12,819 | (1.1%) | 13,329 | (1.1%) | 0.0115 |
| Coagulopathy | 48,972 | (1.1%) | 12,052 | (1.1%) | 12,982 | (1.1%) | 11,967 | (1.1%) | 11,971 | (1.0%) | 0.196 |
| Pulmonary Circulation Disorders | 41,608 | (0.9%) | 9,536 | (0.8%) | 10,385 | (0.9%) | 10,909 | (1.0%) | 10,778 | (0.9%) | 0.0567 |
| Liver Disease | 41,212 | (0.9%) | 9,116 | (0.8%) | 10,311 | (0.9%) | 10,531 | (0.9%) | 11,254 | (0.9%) | 0.0049 |
| Psychoses | 33,974 | (0.7%) | 7,463 | (0.7%) | 8,395 | (0.7%) | 8,792 | (0.8%) | 9,324 | (0.8%) | 0.016 |

| | | | | | | | | | | | |
|---|---|---|---|---|---|---|---|---|---|---|---|
| Weight Loss | 29,585 | (0.6%) | 6,477 | (0.6%) | 7,555 | (0.6%) | 7,679 | (0.7%) | 7,874 | (0.7%) | 0.0094 |
| Metastatic Cancer | 18,150 | (0.4%) | 4,156 | (0.4%) | 4,235 | (0.4%) | 4,781 | (0.4%) | 4,979 | (0.4%) | 0.0155 |
| Lymphoma | 12,141 | (0.3%) | 2,930 | (0.3%) | 2,968 | (0.3%) | 2,808 | (0.2%) | 3,435 | (0.3%) | 0.0632 |
| Paralysis | 6,925 | (0.1%) | 1,627 | (0.1%) | 1,797 | (0.2%) | 1,665 | (0.1%) | 1,836 | (0.2%) | 0.6787 |
| Peptic Ulcer Disease Excluding Bleeding | 6,863 | (0.1%) | 1,961 | (0.2%) | 1,819 | (0.2%) | 1,537 | (0.1%) | 1,546 | (0.1%) | 0.0046 |
| AIDS/HIV | 6,586 | (0.1%) | 1,387 | (0.1%) | 1,771 | (0.2%) | 1,528 | (0.1%) | 1,900 | (0.2%) | 0.2316 |
| Blood Loss Anemia | 5,649 | (0.1%) | 1,242 | (0.1%) | 1,543 | (0.1%) | 1,404 | (0.1%) | 1,460 | (0.1%) | 0.205 |

**Table S4: The Sum of Elixhauser Comorbidity Index score of the Patient Population Stratified by the Calendar Year.**

| | Total (n = 4,645,483) | | 2016 (n = 1,145,359) | | 2017 (n = 1,174,452) | | 2018 (n = 1,137,276) | | 2019 (n = 1,188,396) | |
|---|---|---|---|---|---|---|---|---|---|---|
| 0 | 1,670,288 | (36.0%) | 426,332 | (37.2%) | 421,696 | (35.9%) | 403,301 | (35.5%) | 418,959 | (35.3%) |
| 1 | 1,133,290 | (24.4%) | 281,388 | (24.6%) | 285,393 | (24.3%) | 277,458 | (24.4%) | 289,052 | (24.3%) |
| 2 | 772,046 | (16.6%) | 189,505 | (16.5%) | 196,974 | (16.8%) | 187,604 | (16.5%) | 197,962 | (16.7%) |
| 3 | 484,450 | (10.4%) | 116,117 | (10.1%) | 122,405 | (10.4%) | 119,534 | (10.5%) | 126,395 | (10.6%) |
| 4 | 286,699 | (6.2%) | 66,122 | (5.8%) | 73,210 | (6.2%) | 72,124 | (6.3%) | 75,242 | (6.3%) |
| 5 | 156,813 | (3.4%) | 35,238 | (3.1%) | 39,583 | (3.4%) | 39,911 | (3.5%) | 42,081 | (3.5%) |
| 6 | 80,047 | (1.7%) | 17,418 | (1.5%) | 20,008 | (1.7%) | 20,990 | (1.8%) | 21,631 | (1.8%) |
| 7 | 37,003 | (0.8%) | 8,059 | (0.7%) | 9,217 | (0.8%) | 9,643 | (0.8%) | 10,084 | (0.8%) |
| 8 | 15,777 | (0.3%) | 3,299 | (0.3%) | 3,680 | (0.3%) | 4,366 | (0.4%) | 4,432 | (0.4%) |
| 9 | 6,361 | (0.1%) | 1,357 | (0.1%) | 1,682 | (0.1%) | 1,619 | (0.1%) | 1,703 | (0.1%) |
| 10 | 1,964 | (0.0%) | 377 | (0.0%) | 467 | (0.0%) | 468 | (0.0%) | 652 | (0.1%) |
| 11 | 611 | (0.0%) | 102 | (0.0%) | 122 | (0.0%) | 210 | (0.0%) | 177 | (0.0%) |
| 12 | 92 | (0.0%) | 27 | (0.0%) | 7 | (0.0%) | 32 | (0.0%) | 27 | (0.0%) |
| 13 | 38 | (0.0%) | 14 | (0.0%) | 9 | (0.0%) | 16 | (0.0%) | 0 | (0.0%) |
| 14 | 4 | (0.0%) | 4 | (0.0%) | 0 | (0.0%) | 0 | (0.0%) | 0 | (0.0%) |

The p-value for significance is 0.5889. The Sum Elixhauser Comorbidity Index is presented here as a continuous variable.
